# Down-regulation of SOX18 inhibits laryngeal carcinoma cell proliferation, migration, and invasion through JAK2/STAT3 signaling

**DOI:** 10.1042/BSR20182480

**Published:** 2019-07-05

**Authors:** Yice Xu, Qingyuan Zhang, Jie Zhou, Zhaolong Li, Junyu Guo, Weina Wang, Wei Wang

**Affiliations:** Department of Otolaryngology-Head and Neck Surgery, Xiaogan Central Hospital Affiliated to Wuhan University of Science and Technology, Xiaogan 432000, China

**Keywords:** Invasion, JAK2/STAT3 signaling, Laryngeal carcinoma, Migration, Proliferation, SOX18

## Abstract

Laryngeal carcinoma is one of the most common malignant tumors of the head, neck, and respiratory tract. The aim of the present study is to explore the biological function of SRY-related HMG-box 18 (SOX18) in laryngeal carcinoma cells and study the molecular mechanism involved. Initial findings indicate that the expression of SOX18 was increased in laryngeal carcinoma cell lines and tissues. The effect of SOX18 on laryngeal carcinoma cell proliferation, cell cycle, apoptosis, invasion, and migration was also identified. The results indicated that down-regulation of SOX18 significantly inhibited cell proliferation, migration, and invasion, and induced cell-cycle arrest in G_0_/G_1_ phase and apoptosis of laryngeal carcinoma cells. However, overexpression of SOX18 promoted cell proliferation, invasion, and migration, and inhibited cell apoptosis. The expression of cyclin D1, active-caspase-3, N-cadherin, MTA1, MMP-2, and MMP-7 was also regulated by the overexpression of siSOX18 or SOX18. In addition, it was found that SOX18 could also accelerate the phosphorylation of JAK2/STAT3 signaling in laryngeal carcinoma cells. Furthermore, our study indicated that SOX18 could stimulate cell proliferation, migration, and invasion of laryngeal carcinoma cells via regulation of JAK2/STAT3 signaling, which could provide a new strategy for laryngeal carcinoma diagnosis and molecular therapies.

## Introduction

Laryngeal carcinoma is one of the most common malignant tumors among people with a history of head and neck tumors. The overall incidence of laryngeal cancer has remarkably increased and has become the second most common cancer of respiratory system [1,2]. Most of the laryngeal carcinomas develop from squamous cells and thus referred to as squamous cell carcinomas. Although, advances in medical science and technology has improved the treatment of laryngeal cancer, including surgery, chemotherapy, radiotherapy, and targeted drugs, it could not improve the overall survival rate of patients with laryngeal cancer [3,4]. Improving the early diagnosis rate of laryngeal carcinoma, early prediction of recurrence and effective intervention measures are the key to improve the curative effect of laryngeal carcinoma [5,6]. Advances in molecular medicine has also helped in finding the specific therapeutic target for treatment, thus improving the curative effect of laryngeal cancer [7,8].

SRY-related HMG-box 18 (SOX18) belongs to the family of transcription factors, which is involved in the regulation of embryonic development and in the determination of the cell fate [9]. Recent studies reported that the overexpression of SOX18 was found in various types of tumors such as breast cancer, non-small cell lung cancer, prostate cancer, and non-melanoma skin cancer [10–13], and its expression correlates with cell proliferation, migration, and invasion of tumor cells [14,15]. The role and mechanism of SOX18 in laryngeal carcinoma still remains unclear.

In the present study, we aimed to explore the expression and effect of SOX18 on tumor cell physiology and interpret the molecular mechanism involved. Overexpression of SOX18 was found in the tumor tissues of laryngeal carcinoma patients, which regulated cell proliferation, migration, and invasion of laryngeal carcinoma cells through JAK2/STAT3 signaling.

## Materials and methods

### Patients and tissue samples

Laryngeal cancer tissues and corresponding adjacent non-tumor tissues (located 5 cm away from laryngeal cancer tissues) were obtained from 21 patients in Xiaogan Central Hospital Affiliated to Wuhan University of Science and Technology (Wuhan, China) from November 2013 to May 2015. The information of 21 patients was shown in Table 1. All clinical samples obtained from patients were immediately frozen in liquid nitrogen and stored at −80°C for further studies. The present study was approved by the Ethnics and Scientific Committee of Xiaogan Central Hospital Affiliated to Wuhan University of Science and Technology (Wuhan, China) and was performed in compliance with the Declaration of Helsinki. A written informed consent was obtained from all the patients recruited in the present study.

**Table 1:** Clinical characteristics of patients with laryngeal carcinoma (*n*=21)

Characteristics	*n* (%)
Gender	
Male	13 (61.90%)
Female	8 (38.10%)
Age	
≤60	9 (42.86%)
>60	12 (57.14%)
Grade	
I	3 (14.29%)
II	10 (47.62%)
III	8 (38.10%)
Lymph node	
N0	9 (42.86%)
N1–N3	12 (57.14%)
Stage	
I–II	12 (57.14%)
III–IV	9 (42.86%)

### Immunohistochemistry

Tissue sections were initially deparaffinized, rehydrated, and then heated in EDTA (pH 8.0); antigen retrieval was performed in 10-mM citrate buffer for 5 min at 100°C. The incubation with SOX18 antibody (1:200; cat. no. ab109194, Abcam, Cambridge, MA, U.S.A.) was performed at room temperature for 1 h, followed by incubation with biotin-labeled secondary antibodies. Slides were then developed using DAB solution and counterstained with hematoxylin staining (BASO, Zhuhai, China). Immunohistochemical signals were calculated with positive staining cells under a microscope (Olympus Corporation, Tokyo, Japan) with magnification of 200× and 400×.

### Cell culture

Three human laryngeal cancer cell lines (SNU899, AMC-HN-8, and Hep-2) and one normal human bronchial epithelial cell line (BEAS-2B) were purchased from Shanghai Cell Bank, Chinese Academy of Sciences (Shanghai, China). Cells were cultured in RPMI 1640 medium (Gibco, Los Angeles, CA, U.S.A.) supplemented with 10% fetal bovine serum (FBS) (Sigma–Aldrich, St. Louis, MO, U.S.A.), 100 units/ml penicillin, and 100 μg/ml streptomycin. All the cells were maintained in a humidified incubator with 95% air and a 5% CO_2_ atmosphere at 37°C.

### RNA extraction and quantitative real-time PCR

Total RNA was extracted from tissues and cells using TRIzol reagent (Invitrogen) according to the manufacturer’s instructions, and quantified using Nanodrop 2000 (Thermo Fisher Scientific, Waltham, MA, U.S.A.). For mRNA expression analysis, the first strand was synthesized using TaqMan High-Capacity cDNA Reverse Transcription (Applied Biosystems). Glyceraldehyde 3-phosphate dehydrogenase (GAPDH) was used as an internal control to normalize the expression of SOX18 mRNA. Quantitative real-time PCR (qRT-PCR) was performed using the Applied Biosystems 7500 Fast Real-Time PCR system. The specific primer sequences (Invitrogen) were as follows: SOX18, forward 5′-CGCGTGTATGTTTGGTTC-3 and reverse 5′-ATGTAACCCTGGCAACTC-3′; GAPDH, forward 5′-CACCCACTCCTCCACCTTTG-3′ and reverse 5′-CCACCACCCTGTTGCTGTAG-3′. The relative gene expression levels were calculated using the 2^−ΔΔ*C*^_t_ method.

### Cell transfection

Cell transfection was carried out using Lipofectamine 2000 (Invitrogen, Waltham, U.S.A.) according to manufacturer’s protocol. Cells were transfected with siSOX18-1 or siSOX18-2, siControl (siCtrl), SOX18 or Control (Ctrl). The siRNA against SOX18 was designed and synthesized by Shanghai GenePharma Co., Ltd. (Shanghai, China). Cells were harvested 48 h post-transfection for further experiments.

### Cell viability assay

Cell proliferation was evaluated using MTT Cell Proliferation and Cytotoxicity Assay Kit (Sigma–Aldrich, St. Louis, U.S.A.) according to the manufacturer’s protocol. Briefly, cells were seeded into 96-well plates at a density of 2–3 × 10^3^ cells/well after transfection. It was then incubated at 37°C for different time periods (0, 12, 24, 48, and 72 h). Subsequently, the culture medium was removed and replaced with 100 μl sterile MTT (0.5 mg/ml, Sigma). After incubation at 37°C for another 4 h, MTT solution was removed and replaced with 150 μl dimethyl sulfoxide (DMSO) (4%, Sigma). After incubation with DMSO for 15 min, the absorbance was measured at 450 nm via a microplate reader (Bio-Tek Instrument, Vermont, U.S.A.).

### Cell-cycle distribution analysis

Forty-eight hours post-transfection, cells were harvested. These cells were washed with PBS and fixed in ethanol at −20˚C. The cells were again washed with PBS, rehydrated, and resuspended in propidium iodide (PI; 10 µl)-RNase A solution (Sigma–Aldrich; Merck KGaA) at 37˚C for 30 min. The stained cells (1 × 10^5^) were then analyzed for DNA content using a flow cytometer (BD Biosciences, Franklin Lakes, NJ, U.S.A.). The results were analyzed using FlowJo 7.6.1 software (FlowJo LLC, Ashland, OR, U.S.A.).

### Cell apoptosis detection

Cells were harvested, washed with ice-cold PBS, and stained with Annexin V-FITC apoptosis detection kits (Nanjing KeyGen Biotech Co., Ltd., Nanjing, China). Cell apoptosis was analyzed using FlowJo 7.6.1, a software used to analyze flow cytometry data (BD Biosciences).

### Cell migration and invasion assay

Cell migration was performed in a chamber having a filter membrane of 8 μm pore-size (Corning, New York, NY, U.S.A.). Cells were grown to ∼50% confluency and transfected with SOX18 siRNA. Twenty-four hours post transfection, the cells were incubated in serum-free medium for 24 h. Cells were then trypsinized and 5 × 10^4^ cells in serum-free medium were added to the upper chamber. Media with 10% FBS was then added to the lower chamber. After incubation at 37°C for 24 h, non-migrating cells were completely removed. Cells that migrated to the bottom of the membrane were then fixed in 4% paraformaldehyde and stained by 0.5% crystal violet. Stained cells were visualized under a microscope and average number of migrated cells in five random fields were counted.

For invasion assay, the upper chamber was precoated with 1 mg/ml Matrigel (BD Biosciences). The rest of the procedure is similar to the cell-migration assay.

### Western blot analysis

Proteins were extracted from cells using the protein extraction reagent (Takara, Dalian, China). The BCA Protein Assay Kit (Takara) was applied to detect the concentrations of the extracted proteins. The extracts were separated by using 10% sodium dodecyl sulfate/polyacrylamide gel electrophoresis (SDS/PAGE) and transferred onto polyvinylidene fluoride (PVDF) microporous membranes (Dupont NEN, Boston, MA, U.S.A.). The PVDF membranes were blocked with PBS containing 0.1% Tween-20 (PBST) and 5% (w/v) skimmed milk for 1 h at room temperature. After washing it for three times with PBST, the PVDF membranes were probed with corresponding antibodies overnight at 4°C. Anti-cyclin D1 (ab134175), anti-cleaved-caspase-3 (ab2302), anti-caspase-3 (ab13585), anti-N-cadherin (ab18203), anti-MTA1 (ab71153), anti-MMP-2 (ab37150), anti-MMP-7 (ab207299) anti-PDGF (ab178409), anti-IGF1R (ab39398), anti-JAK2 (ab108596), anti-phosphorylation-JAK2 (ab32101), anti-STAT3 (ab119352), anti-phosphorylation-STAT3 (ab76315), and anti-GAPDH (ab8245) were purchased from Abcam (Cambridge, MA, U.S.A.) and the dilutions used are as follows: anti-cyclin D1 (1:800), anti-cleaved-caspase-3 (1:800), anti-caspase-3 (1:800), anti-N-cadherin (1:1000), anti-MTA1 (1:800), anti-MMP2 (1:1000), anti-MMP-7 (1:1000), anti-PDGF (1:800), anti-IGF1R (1:800), anti-JAK2 (1:1000), anti-phosphorylation-JAK2 (1:1000), anti-STAT3 (1:1000), anti-phosphorylation-STAT3 (1:1000), and anti-GAPDH (1:3000). After the PVDF membranes were washed again with PBST, horseradish peroxidase (HRP)-conjugated IgG was added at 1:5000 dilution and incubated at room temperature for 1 h. The blots were developed using enhanced chemiluminescence (ECL) Western blotting detection reagents.

### Statistical analysis

Data were represented as the mean ± standard deviation (SD) of three separate experiments. Statistical analysis was performed using SPSS 18.0 (SPSS, Chicago, IL, U.S.A.). Two-tailed student’s *t* test was applied to compare the differences between two groups and one-way analysis of variance (ANOVA) followed by Dunnett’s multiple comparison was employed to compare the differences between three or more independent groups. The result is considered statistically significant if *P*<0.05.

## Results

### Increased expression of SOX18 in laryngeal carcinoma cell lines and tissues

**Figure 1:**
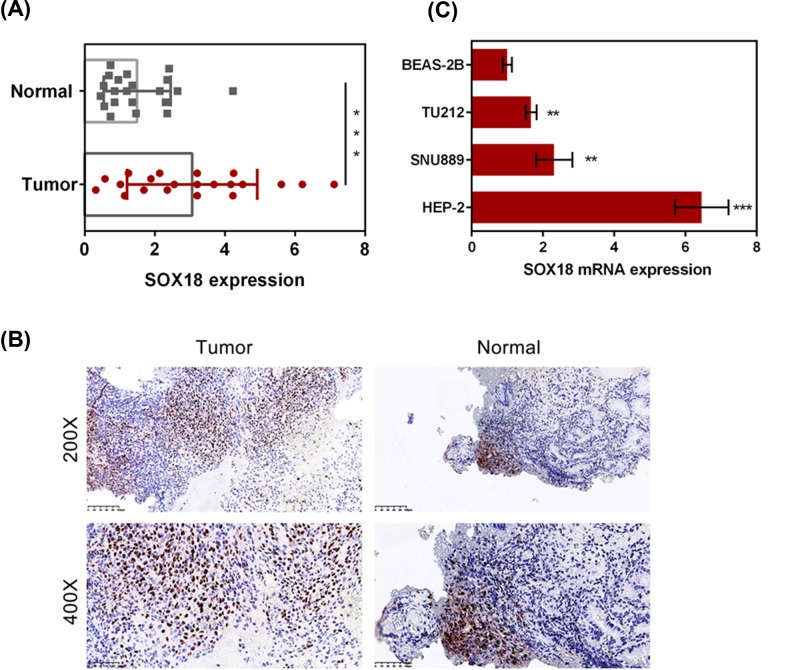
SOX18 expression in laryngeal carcinoma cell lines and tissues (**A,B**) The expression of SOX18 in the tissues of laryngeal carcinoma patients and normal tissues was identified by RT-PCR and immunohistochemical analysis. (**C**) The expression of SOX18 in laryngeal cancer cell lines (SNU899, AMC-HN-8, and Hep-2) and normal human bronchial epithelial cell line (BEAS-2B) was detected by RT-PCR. Data were represented as mean ± SD. *P<0.05, **P<0.01 and ***P<0.001 versus Normal or BEAS-2B cell line.

To evaluate the role of SOX18 in laryngeal carcinoma, the expression of SOX18 in laryngeal carcinoma cell lines and tissues were determined by qRT-PCR and immunohistochemical analysis. As shown in Figure 1A,B, an increase in SOX18 level was observed in laryngeal carcinoma tissues compared with the adjacent normal tissues (*P*<0.01). Furthermore, mRNA expression of SOX18 in laryngeal cancer cell lines (SNU899, TU212, and Hep-2) and normal bronchial epithelial cell line (BEAS-2B) were also evaluated by qRT-PCR. The expression of SOX18 was significantly increased in laryngeal cancer cell lines compared with that in BEAS-2B cells (*P*<0.01, Figure 1C). Hep-2 and TU212 cell lines were then analyzed for further studies. The results showed that SOX18 might act as an oncogene in the occurrence and progression of laryngeal carcinoma.

### SOX18 regulated cell proliferation, cell cycle, and cell apoptosis in laryngeal carcinoma cell lines

**Figure 2:**
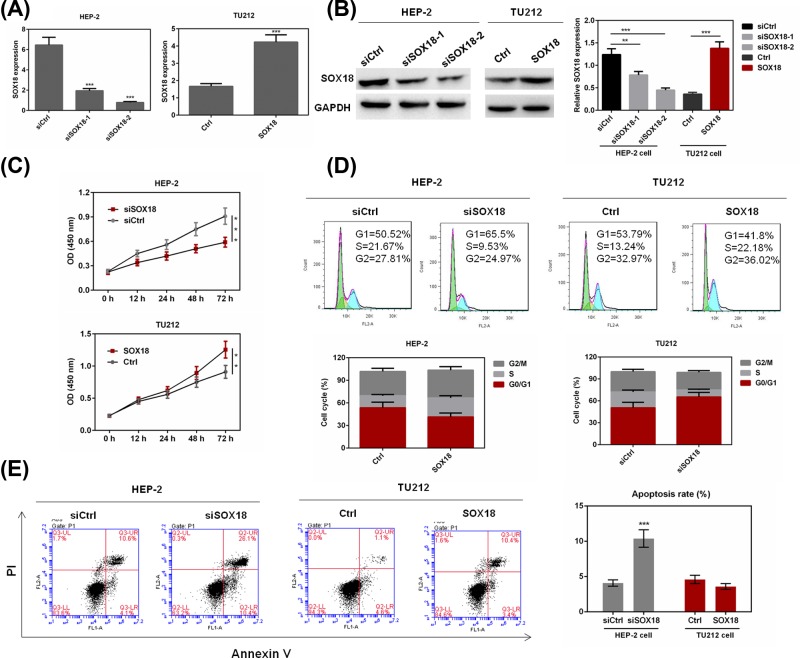
The effect of SOX18 expression on cell proliferation, cell cycle, and cell apoptosis of laryngeal carcinoma cells (**A,B**) Hep-2 cells were transfected with siSOX18 or siCtrl, and TU212 cells were transfected with SOX18 or Ctrl. The transfection efficiency was examined by RT-PCR and Western blot. (**C**) Cell proliferation of Hep-2 and TU212 cells transfected with siSOX18 or SOX18 was determined by MTT assay. (**D**) Cell-cycle distribution of Hep-2 and TU212 cells transfected with siSOX18 or SOX18 was determined by flow cytometry. (**E**) Cell apoptosis of Hep-2 and TU212 cells transfected with siSOX18 or SOX18 was evaluated by Annexin V/PI staining. Data were represented as mean ± SD. *P<0.05, **P<0.01 and ***P<0.001 versus siCtrl or Ctrl group.

To identify the role of SOX18 in laryngeal carcinoma cell functions, Hep-2 was transfected with siSOX18 and TU212 was transfected with SOX18-overexpression plasmid. Two siRNAs were conducted for SOX18 knockdown, and the transfection efficiency was examined by qRT-PCR and Western blot analysis (Figure 2A,B). The siSOX18-2 exhibited better effect and was carried out for further studies. Cell proliferation was then detected by MTT assay. The results showed that down-regulation of SOX18 significantly suppressed cell viability in Hep-2 cells, while overexpression of SOX18 drastically promoted cell proliferation in TU212 cells (*P*<0.05, Figure 2C). Cell cycle was then evaluated by flow cytometry, and the results showed that silencing the expression of SOX18 could notably induce G_0_/G_1_ phase arrest in Hep-2 cells and up-regulation of SOX18 could increase the cell count in S and G_2_ phase in TU212 cells (*P*<0.05, Figure 2D). Furthermore, cell apoptosis was examined by immunostaining with Annexin V/PI. As shown in Figure 2E, cell apoptosis was promoted effectively by siSOX18 transfection in Hep-2 cells (*P*<0.01) and was inhibited by the overexpression of SOX18 in TU212 cells. The above results indicate that SOX18 could regulate cell growth and apoptosis in laryngeal carcinoma cells.

### Knockdown of SOX18 suppressed cell migration and invasion in laryngeal carcinoma cell lines

**Figure 3:**
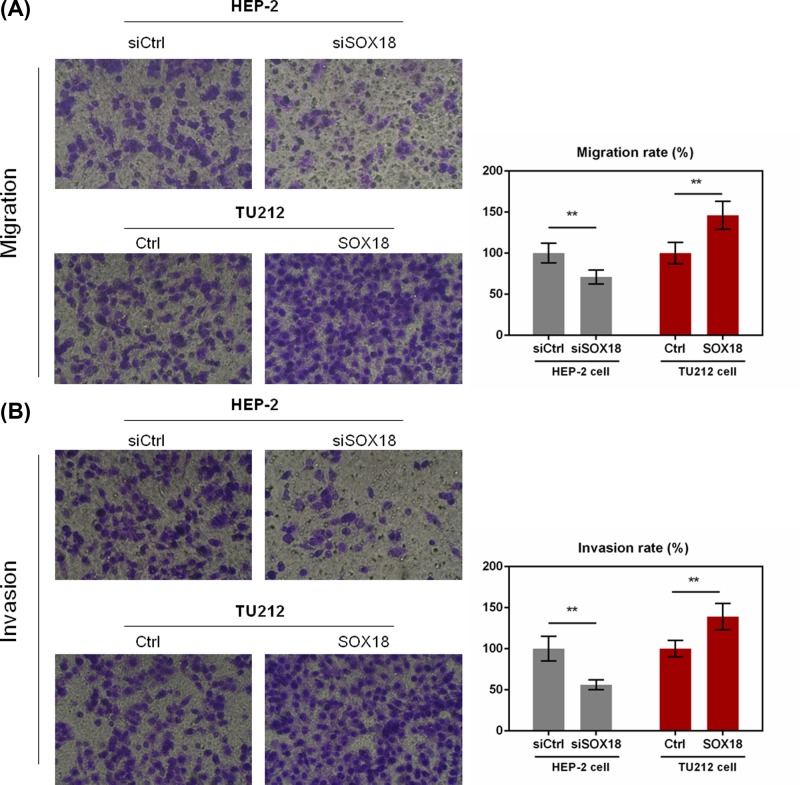
The effect of SOX18 expression on cell migration and invasion of laryngeal carcinoma cells (**A,B**) Cell migration and invasion of Hep-2 and TU212 cells transfected with siSOX18 or SOX18 were evaluated by transwell assay (magnification 100×). Data were presented as mean ± SD. **P<0.01 versus siCtrl or Ctrl group.

To further identify the role of SOX18 in metastasis of laryngeal carcinoma, the effects of SOX18 on cell migration and invasion were evaluated by transwell assay. As shown in Figure 3A, SOX18 knockdown significantly weakened cell migration and overexpression of SOX18 remarkably increased cell migration in laryngeal carcinoma cell lines (*P*<0.01). For laryngeal carcinoma cell invasion, siSOX18 could notably inhibit cell invasion, and up-regulation of SOX18 could significantly promote cell invasion in laryngeal carcinoma cell lines (*P*<0.01, Figure 3B). The results revealed that SOX18 could function as an oncogene in laryngeal carcinoma.

### SOX18 regulated cell proliferation and expression of invasion-related proteins

**Figure 4:**
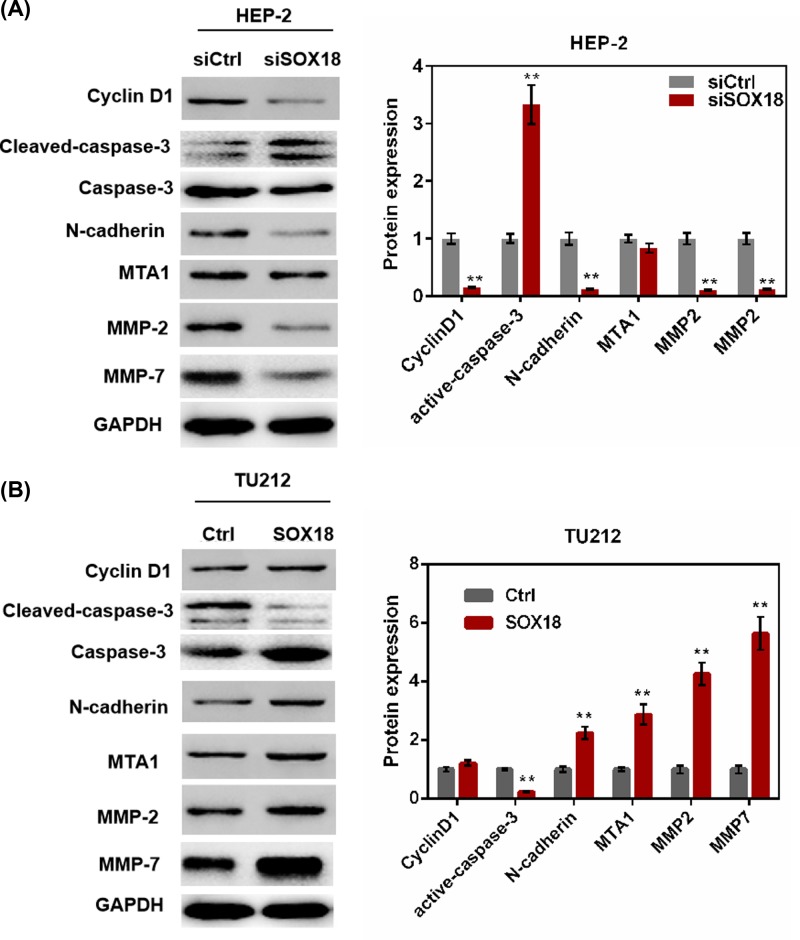
SOX18 regulated the expression of cyclin D1, active-caspase-3, N-cadherin, MTA1, MMP-2, and MMP-7 (**A,B**) The expression of cyclin D1, active-caspase-3, N-cadherin, MTA1, MMP-2, and MMP-7 of Hep-2 and TU212 cells transfected with siSOX18 or SOX18 was detected by Western blot assay. Data were represented as mean ± SD. **P<0.01 versus siCtrl or Ctrl group.

To explore the mechanism by which SOX18-regulated cell functions in laryngeal carcinoma, the expression of Cyclin D1, cleaved-caspase-3/caspase-3, N-cadherin, MTA1, MMP-2, and MMP-7 were detected by Western blot analysis. In Figure 4A, down-regulation of SOX18 in Hep-2 cells could decrease the expression of Cyclin D1, N-cadherin, MMP-2, and MMP-7, and increase the expression of active-caspase-3 (*P*<0.01). Up-regulation of SOX18 in TU212 cells could significantly decrease the expression of active-caspase-3 and increase MTA1, N-cadherin, MMP-2, and MMP-7 expression (*P*<0.01, Figure 4B). The results demonstrated that SOX18 could regulate cell activities via regulating the expression of cyclin D1, active-caspase-3, N-cadherin, MTA1, MMP-2, and MMP-7.

### SOX18 regulated the JAK2/STAT3 signaling in laryngeal carcinoma cell lines

**Figure 5:**
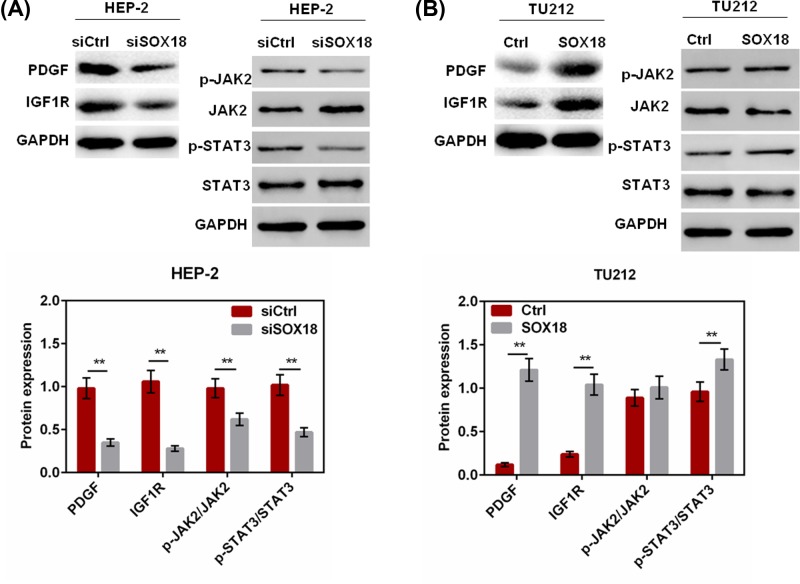
SOX18 regulated the phosphorylation of JAK2/STAT3 signaling (**A,B**) The expressions of PDGF and IGF1R, and phosphorylation of JAK2 and STAT3 in Hep-2 and TU212 cells transfected with siSOX18 or SOX18 was detected by Western blot assay. Data were represented as mean ± SD. **P<0.01 versus siCtrl or Ctrl group.

To further evaluate the regulation of cell proliferation, apoptosis, migration, and invasion of laryngeal cancer cells by SOX18, Western blot analysis was used to detect the expressions of JAK-STAT3 upstream effectors (PDGF and IGF1R), and the phosphorylation of JAK2 and STAT3. We found that siSOX18 effectively inhibited the expressions of PDGF and IGF1-R, while SOX18 overexpression promoted expressions of PDGF and IGF1-R (*P*<0.01, Figure 5A,B). In addition, the results showed that SOX18 knockdown in Hep-2 cells significantly inhibited the phosphorylation of JAK2 and STAT3 (*P*<0.01, Figure 5A). On the contrary, overexpression of SOX18 effectively accelerated the phosphorylation of JAK2 and STAT3 in TU212 cells (*P*<0.01, Figure 5B). The results revealed that SOX18 promoted carcinogenesis in laryngeal carcinoma via activating JAK2/STAT3 signaling.

## Discussion

Laryngeal cancer is the most common malignant tumor in the department of otorhinolaryngology and ranked second among the head and neck primary malignant tumors, which are of epithelial origin. Effective screening and use of sensitive biomarkers of laryngeal cancer is crucial for laryngeal cancer diagnosis and treatment. Previous studies indicated that SOX18 exhibited oncogenic activity in various types of cancers. Jethon et al*.* [13] indicated that expression of SOX18 correlated with poor patient outcome in non-small cell lung cancer. Yin et al. [10] reported that the expression of SOX18 was increased in prostate cancer and regulated the malignant capacity of prostate cancer cells via regulation of TCF1, c-Myc, cyclin D1, and MMP-7 expression. Zhu et al. demonstrated that SOX18 was overexpressed in osteosarcoma patients and interacted with HERC2 to regulate cell malignant phenotypes of osteosarcoma cells [14]. In the present study, laryngeal cancer tissues and adjacent normal tissues were collected for the detection of SOX18 expression. It was initially found that the expression of SOX18 was increased drastically in laryngeal cancer tissues compared with normal tissues. SOX18 was also overexpressed in laryngeal cancer cell lines. The findings revealed that SOX18 was a critical regulator, which is involved in human laryngeal cancer.

Cell malignant phenotypes, including abnormal cell proliferation and decreased cell apoptosis play a crucial role in tumorigenesis. Wang et al. [16] proved that SOX18 promoted cell proliferation and reduced cell apoptosis in pancreatic ductal adenocarcinoma cells. Zhang et al*.* showed that knockdown of SOX18 significantly inhibited cell proliferation and invasion, but promoted apoptosis in breast cancer cells [15]. In the present study, we found that down-regulation of SOX18 significantly inhibited cell proliferation, induced cell-cycle arrest in G_0_/G_1_ phase and cell apoptosis in Hep-2 cells. While up-regulation of SOX18 notably promoted cell proliferation, increased the cell distribution in S and G_2_ phase, and decreased cell apoptosis in TU212 cells. The effect of SOX18 on cell migration and invasion was also examined. The results showed that siSOX18 effectively decreased cell migration and invasion capability, and SOX18 could promote cell migration and invasion in laryngeal carcinoma cells. Furthermore, it was found that SOX18 may also promote the occurrence and development of laryngeal carcinoma.

The effect of SOX18 on the expression of oncogenic proteins and tumor suppressors was also examined. Cyclin D1 can activate CDK4 in G_1_ phase and exerts proliferative actions [17]. Increased expression of cleaved-caspase-3 promotes cell apoptosis [18]. Excessive expressions of N-cadherin, MTA1, MMP-2, and MMP-7 also facilitate tumor metastasis [19–21]. In our study, SOX18 knockdown effectively inhibited the expression of cyclin D1, N-cadherin, MTA1, MMP-2, and MMP-7, and increased the expression of active caspase-3. However, up-regulation of SOX18 showed the opposite effect. To further explore the possible mechanism by which SOX18 regulated cell functions in laryngeal cancer, the effect of SOX18 on JAK2/STAT3 signaling was examined. JAK2/STAT3 signal pathway exists in various types of cancers including laryngeal cancer [22,23], which is correlated with cell proliferation, apoptosis, and differentiation [24,25]. In our study, the results indicated that down-regulation of SOX18 significantly suppressed the expressions of JAK-STAT3 upstream effectors (PDGF and IGF1R) and the phosphorylation of JAK2 and STAT3, while overexpression of SOX18 exerted the contrary effect. These findings revealed that SOX18 could regulate cell proliferation, cell cycle, apoptosis, migration, and invasion of laryngeal carcinoma cells via JAK2/STAT3 signaling.

Consequently, we demonstrated that SOX18 was overexpressed in laryngeal carcinoma cell lines and tissues, and regulated the cell proliferation, cell cycle, apoptosis, migration, and invasion of laryngeal carcinoma cells via JAK2/STAT3 signaling. To conclude, our research suggested that targeting SOX18 might provide a novel treatment strategy for laryngeal carcinoma.

## Abbreviations

FBS: fetal bovine serum
DMSO: dimethyl sulfoxide
GAPDH: glyceraldehyde 3-phosphate dehydrogenase
IGF1R: Insulin-like growth factor 1 receptor
JAK: Janus kinase
MMP-2: Matrix metalloproteinase-2
MTA1: Matastasis associated protein 1
PBST: Phosphate buffered saline
PVDF: polyvinylidene fluoride
qRT-PCR: quantitative real-time PCR
SD: standard deviation
SiCtrl: SiControl
SOX18: SRY-related HMG-box 18
STAT3: Signal transducer and activator of transcription

## References

[1] Sahli M., Hemmaoui B. and Benariba F. (2017) Intramedullary spinal cord metastasis from laryngeal carcinoma: case report and review of literature. Pan Afr. Med. J. 26, 189 10.11604/pamj.2017.26.189.11507 28674582 PMC5483368

[2] Xia Y., Qi C., Zhang S., Huang Z. and Chen Y. (2017) Elevated 18F-NaF uptake in cricoid cartilage in a patient with laryngeal carcinoma: a case report and literature review. Medicine 96, e9090 10.1097/MD.0000000000009090 29245332 PMC5728947

[3] Wong W.K. and Vokes D. (2018) Solitary femoral metastasis in a locoregionally controlled laryngeal carcinoma. ANZ J. Surg. 10.1111/ans.14403 29761605

[4] Bozan N., Demir H., Gursoy T., Ozkan H., Duzenli U., Sarikaya E. (2018) Alterations in oxidative stress markers in laryngeal carcinoma patients. J. Chin. Med. Assoc., 81, 811–815 10.1016/j.jcma.2018.02.004 29778552

[5] Zhang X. and Wu N. (2018) Fasudil inhibits proliferation and migration of Hep-2 laryngeal carcinoma cells. Drug Des. Dev. Ther. 12, 373–381 10.2147/DDDT.S147547 29503530 PMC5825979

[6] Shen X., Gao X., Li H., Gu Y. and Wang J. (2018) TIMP-3 increases the chemosensitivity of laryngeal carcinoma to cisplatin via facilitating mitochondria-dependent apoptosis. Oncol. Res., 27, 73–80 10.3727/096504018X15201099883047 29523219 PMC7848409

[7] Yilmaz B., Sengul E., Gul A., Alabalik U., Ozkurt F.E., Akdag M. (2018) Neutrophil-lymphocyte ratio as a prognostic factor in laryngeal carcinoma. Indian J. Otolaryngol. Head Neck Surg. 70, 175–179 10.1007/s12070-014-0769-4 29977836 PMC6015577

[8] Yuan Z., Xiu C., Song K., Pei R., Miao S., Mao X. (2018) Long non-coding RNA AFAP1-AS1/miR-320a/RBPJ axis regulates laryngeal carcinoma cell stemness and chemoresistance. J. Cell. Mol. Med., 22, 4253–4262 10.1111/jcmm.13707 29971915 PMC6111816

[9] Pula B., Olbromski M., Wojnar A., Gomulkiewicz A., Witkiewicz W., Ugorski M. (2013) Impact of SOX18 expression in cancer cells and vessels on the outcome of invasive ductal breast carcinoma. Cell Oncol. 36, 469–483 10.1007/s13402-013-0151-7 24065215 PMC13007479

[10] Yin H., Sheng Z., Zhang X., Du Y., Qin C., Liu H. (2017) Overexpression of SOX18 promotes prostate cancer progression via the regulation of TCF1, c-Myc, cyclin D1 and MMP-7. Oncol. Rep. 37, 1045–1051 10.3892/or.2016.5288 27922675

[11] Overman J., Fontaine F., Moustaqil M., Mittal D., Sierecki E., Sacilotto N. (2017) Pharmacological targeting of the transcription factor SOX18 delays breast cancer in mice. Elife 6, pii: e21221 10.7554/eLife.21221 28137359 PMC5283831

[12] Ornat M., Kobierzycki C., Grzegrzolka J., Pula B., Zamirska A., Bieniek A. (2016) SOX18 expression in non-melanoma skin cancer. Anticancer Res. 36, 2379–2383 27127146

[13] Jethon A., Pula B., Olbromski M., Werynska B., Muszczynska-Bernhard B., Witkiewicz W. (2015) Prognostic significance of SOX18 expression in non-small cell lung cancer. Int. J. Oncol. 46, 123–132 10.3892/ijo.2014.2698 25310193

[14] Zhu D., Yang D., Li X. and Feng F. (2018) Heterogeneous expression and biological function of SOX18 in osteosaroma. J. Cell. Biochem. 119, 4184–4192 10.1002/jcb.26635 29266413

[15] Zhang J., Ma Y., Wang S., Chen F. and Gu Y. (2016) Suppression of SOX18 by siRNA inhibits cell growth and invasion of breast cancer cells. Oncol. Rep. 35, 3721–3727 10.3892/or.2016.4746 27108946

[16] Wang Y., Guo H., Zhang D., Yu X., Leng X., Li S. (2016) Overexpression of SOX18 correlates with accelerated cell growth and poor prognosis in human pancreatic ductal adenocarcinoma. Biochem. Biophys. Res. Commun. 479, 510–516 10.1016/j.bbrc.2016.09.099 27663663

[17] Marampon F., Gravina G.L., Ju X., Vetuschi A., Sferra R., Casimiro M.C. (2016) Cyclin D1 silencing suppresses tumorigenicity, impairs DNA double strand break repair and thus radiosensitizes androgen-independent prostate cancer cells to DNA damage. Oncotarget 7, 64526 28042139 10.18632/oncotarget.12267 PMC5325460

[18] Napso T. and Fares F. (2014) Zebularine induces prolonged apoptosis effects via the caspase-3/PARP pathway in head and neck cancer cells. Int. J. Oncol. 44, 1971–1979 10.3892/ijo.2014.2386 24728469

[19] Hsu C.C., Huang S.F., Wang J.S., Chu W.K., Nien J.E., Chen W.S. (2016) Interplay of N-Cadherin and matrix metalloproteinase 9 enhances human nasopharyngeal carcinoma cell invasion. BMC Cancer 16, 800 10.1186/s12885-016-2846-4 27737648 PMC5064931

[20] Wang R.A. (2014) MTA1–a stress response protein: a master regulator of gene expression and cancer cell behavior. Cancer Metastasis Rev. 33, 1001–1009 10.1007/s10555-014-9525-1 25332145 PMC4244563

[21] Ricci S., Bruzzese D. and DI Carlo A. (2015) Evaluation of MMP-2, MMP-9, TIMP-1, TIMP-2, NGAL and MMP-9/NGAL complex in urine and sera from patients with bladder cancer. Oncol. Lett. 10, 2527–2532 10.3892/ol.2015.3558 26622883 PMC4580016

[22] Zhang H., Zhang D., Luan X., Xie G. and Pan X. (2010) Inhibition of the signal transducers and activators of transcription (STAT) 3 signalling pathway by AG490 in laryngeal carcinoma cells. J. Int. Med. Res. 38, 1673–1681 10.1177/147323001003800512 21309481

[23] Hu A., Huang J.J., Jin X.J., Li J.P., Tang Y.J., Huang X.F. (2015) Curcumin suppresses invasiveness and vasculogenic mimicry of squamous cell carcinoma of the larynx through the inhibition of JAK-2/STAT-3 signaling pathway. Am. J. Cancer Res. 5, 278–288 25628937 PMC4300723

[24] Chen L., Zhou D., Liu Z., Huang X., Liu Q., Kang Y. (2018) Combination of gemcitabine and erlotinib inhibits recurrent pancreatic cancer growth in mice via the JAK-STAT pathway. Oncol. Rep. 39, 1081–1089 29328487 10.3892/or.2018.6198 PMC5802029

[25] Lui A.J., Geanes E.S., Ogony J., Behbod F., Marquess J., Valdez K. (2017) IFITM1 suppression blocks proliferation and invasion of aromatase inhibitor-resistant breast cancer in vivo by JAK/STAT-mediated induction of p21. Cancer Lett. 399, 29–43 10.1016/j.canlet.2017.04.005 28411130 PMC5530765

